# Kasha’s Rule and Koopmans’ Correlations for Electron Tunnelling through Repulsive Coulomb Barriers in a Polyanion

**DOI:** 10.1021/acs.jpclett.2c02145

**Published:** 2022-08-16

**Authors:** Jemma A. Gibbard, Jan R. R. Verlet

**Affiliations:** Department of Chemistry, Durham University, Durham DH1 3LE, United Kingdom

## Abstract

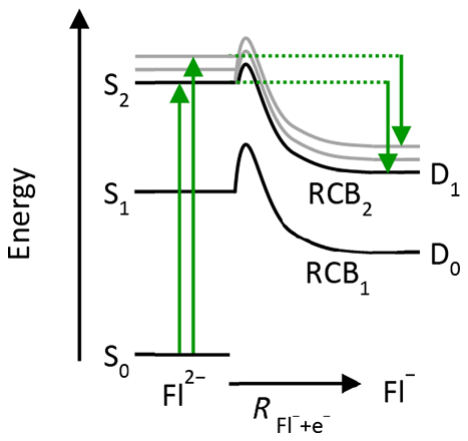

The long-range electronic structure of polyanions is defined by the repulsive Coulomb barrier (RCB). Excited states can decay by resonant electron tunnelling through RCBs, but such decay has not been observed for electronically excited states other than the first excited state, suggesting a Kasha-type rule for resonant electron tunnelling. Using action spectroscopy, photoelectron imaging, and computational chemistry, we show that the fluorescein dianion, Fl^2–^, partially decays through electron tunnelling from the S_2_ excited state, thus demonstrating anti-Kasha behavior, and that resonant electron tunnelling adheres to Koopmans’ correlations, thus disentangling different channels.

The long-range electronic structure of isolated polyanions is characterized by the repulsive Coloumb barrier (RCB) that arises from the balance of long-range repulsive and short-range attractive forces.^1−5^ While often depicted as a single barrier, in reality, the RCB depends on the nuclear and electronic structure of the molecule. There is an RCB associated with each (ro)vibrational level of an electronic state, and each electronic state has its own manifold of RCBs.^6,7^ Photoelectron spectroscopy, and in particular, photoelectron imaging, is well-suited to probing RCBs of polyanions.^6−10^ Photoelectron spectra are generally characterized by a cutoff in the electron kinetic energy (eKE), below which no photoelectrons are emitted, and therefore offers a direct measure of the ground state RCB height. Resonant electron tunnelling through an RCB reports on the excited state and on the pathway linking the vibrational levels of a polyanion to those of the final state with one less electron.^11−20^ Hence, photoelectron spectra of resonant tunnelling give insight into the nature of the excited state RCB. The resonant tunnelling spectra typically show photoelectron emission with an eKE distribution that is independent of photon energy and yields a measure of the energy difference between the mediating resonance and the final state.^11,12^ Resonant electron tunnelling through RCBs of the lowest-lying excited states, S_1_ and T_1_, have been observed.^11−20^ Suprisingly, however, tunnelling emission from higher-lying electronic states has, to the best of our knowledge, not been observed, suggesting that there is a Kasha-type rule^21^ for electron emission by tunnelling in polyanions. In addition there are questions about the applicability of Koopmans’ correlations, which have been used to interpret the photoelecton spectra of excited states, to polyanions.^22,23^ Here, we show resonant tunnelling through the RCB of higher-lying electronic states, demonstrating non-Kasha behavior and that the general Koopmans’ correlations hold for electron tunnelling.

Among the earliest and clearest examples of resonant tunnelling have been the studies on the doubly deprotonated fluorescein dianion (Fl^2–^) and the bisdisulizole tetra-anion.^11,12^ Both studies showed a photoelectron peak with a fixed eKE distribution that was independent of photon energy. For Fl^2–^, the photon energy range only covered the S_1_ ← S_0_ transition,^11^ while for bisdisulizole, it covered >4 eV, suggesting multiple excited states were energetically accessible.^12^ Kasha’s rule pertains to a propensity for fluorescence from the lowest-lying excited state,^21^ which can be consolidated by the fact that internal conversion (IC) between close-lying electronic states is typically faster than fluorescence in complex molecules. The photoelectron spectra of bisdisulizole are therefore consistent with an electron tunnelling analogue of Kasha’s rule, where photoexcitation to a high-lying excited state is followed by a series of rapid IC processes to the S_1_ state, from where the electron ultimately tunnels. The analogous electron tunneling Kasha’s rule in polyanions would depend on the competition between IC and electron emission by tunnelling through the RCBs. The latter can be much faster (e.g., ∼1 ps for tunnelling through the S_1_ RCB in fluorescein)^11^ than fluoresence, raising the general question of whether the resonant electron tunnelling through higher-lying excited state RCBs can occur or whether Kasha’s rule is followed.

We focus on the S_2_ state of Fl^2–^ here. The S_1_ state has been very well characterized. Fl^2–^ fluoresces strongly in aqueous solution following excitation to S_1_ near 500 nm.^24^ In contrast, the S_1_ state of isolated Fl^2–^ decays by electron emission, which allowed Jockusch and co-workers to record an electronic action (absorption) spectrum of the S_1_ ← S_0_ transition.^25,26^ Additionally, our group has previously perfomed photoelectron imaging following excitation to the S_1_ state, which produced a photoelectron spectrum peaking at eKE = 1.64 eV, regardless of the photon energy.^11^ We now extend both these studies to probe the S_2_ state in the UV spectral range.

Frequency-resolved photoelectron imaging was performed as well as mass-resolved fragment action spectroscopy following irradiation of Fl^2–^ with nanosecond (ns) laser light. The photoelectron imaging apparatus has been described previously elsewhere.^27,28^ Modifications have been made to allow the fragment mass and action spectra to be recorded. Fl^2–^ was formed via electrospray ionization of a 5 mM solution of disodium fluorescein salt (Sigma-Aldrich) in methanol. Anions were transferred to a vacuum, and Fl^2–^ was mass-selected using a time-of-flight spectrometer,^29^ the temporal focus of which coincided with the interaction region of a velocity map imaging spectrometer,^30^ where Fl^2–^ was excited. Tunable UV was produced by a Nd:YAG pumped optical parametric oscillator (OPO). Raw photoelectron images were deconvoluted using the polar onion peeling algorithm,^31^ and the resulting photoelectron spectra were calibrated using the spectrum of iodide. Photodetachment of Fl^2–^ also produced the Fl^–^ anion, which was separated using a reflectron secondary mass spectrometer.^32^ The fragment action spectrum was recorded by measuring the yield of Fl^–^ as a function of the laser wavelength from 300 to 400 nm (UV) and 400 to 600 nm (visible). Supporting electronic structure calculations were performed on both Fl^2–^ and Fl^–^ using density functional theory (DFT) for ground states and time-dependent DFT with the Tamm–Dancoff approximation for the excited states, at the B3LYP level of theory with the 6-311G ++ 2d,2p basis set.^33,34^ The Gaussian 16 software package was used.^35^

Figure 1 shows a Fl^2–^ fragment action spectrum in the range λ = 600–300 nm (2.07–4.13 eV). Note that the dashed line at 400 nm (3.10 eV) highlights different ranges of the light source used, and the relative intensities on either side are not comparable. Figure 1 shows a peak at λ = 507 nm that agrees with previous measurements for the S_1_ ← S_0_ transition.^25^ At λ = 325 nm, a second peak is seen that corresponds to excitation of the S_2_ ← S_0_ transition.

**Figure 1:**
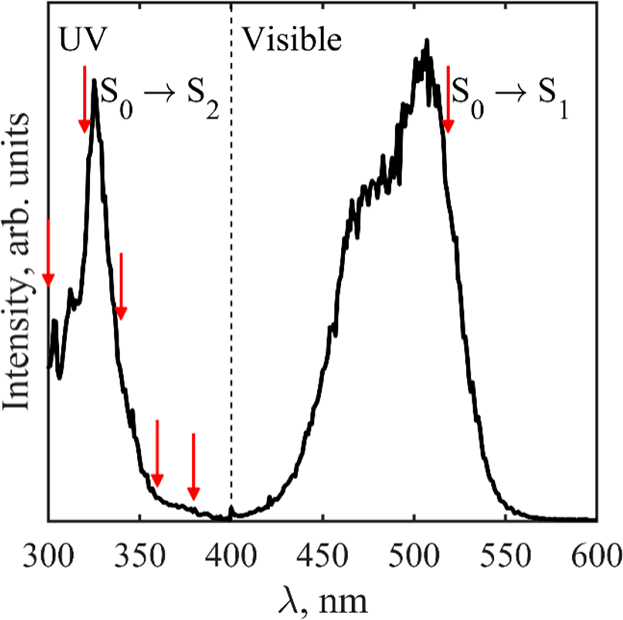
Fl^–^ fragment action spectrum of Fl^2–^ recorded in the range λ = 300–600 nm. The spectrum below 400 nm (UV), as indicated by a dashed line, has been scaled to a similar intensity as above 400 nm (visible) for clarity. The red arrows indicate the wavelengths where photoelectron images have been acquired.

Figure 2 shows a series of photoelectron spectra of Fl^2–^ recorded in the range spanning the S_2_ ← S_0_ transition (specific λ at which the spectra were taken are highlighted with arrows in Figure 1). Additionally, the photoelectron spectrum at λ = 520 nm is shown: this spectrum is representative of *all* spectra taken in the range 460 ≤ λ ≤ 540, where the excitation is resonant with the S_1_ ← S_0_ transition.^11^ The photoelectron spectra have been plotted in terms of eKE to highlight that certain features are invariant with λ. Three features are observed: (i) a peak centered at a fixed eKE = 1.64 eV (highlighted red); (ii) a rising edge at a fixed eKE = 2.2 eV for λ ≤ 340 nm (highlighted green); and (iii) a peak that shifts to higher eKE with *hν* (i.e., fixed electron binding energy, eBE = *hν* – eKE ≈ 0.7 eV, highlighted in blue). The latter corresponds to direct detachment Fl^2–^(S_0_) + *hv* → Fl^–^(D_0_) + e^–^, which was also observed in our previous study for 400 ≤ λ ≤ 440 nm.^11^ From this feature, a vertical detachment energy (VDE) of ∼0.7 eV and an adiabatic detachment energy (ADE) of ∼0.5 eV can be extracted for Fl^2–^. The direct detachment process, which leads to the D_0_ anion state, is depicted in Figure 3a, and its shape depends on the S_0_/D_0_ Franck–Condon factors.

**Figure 2:**
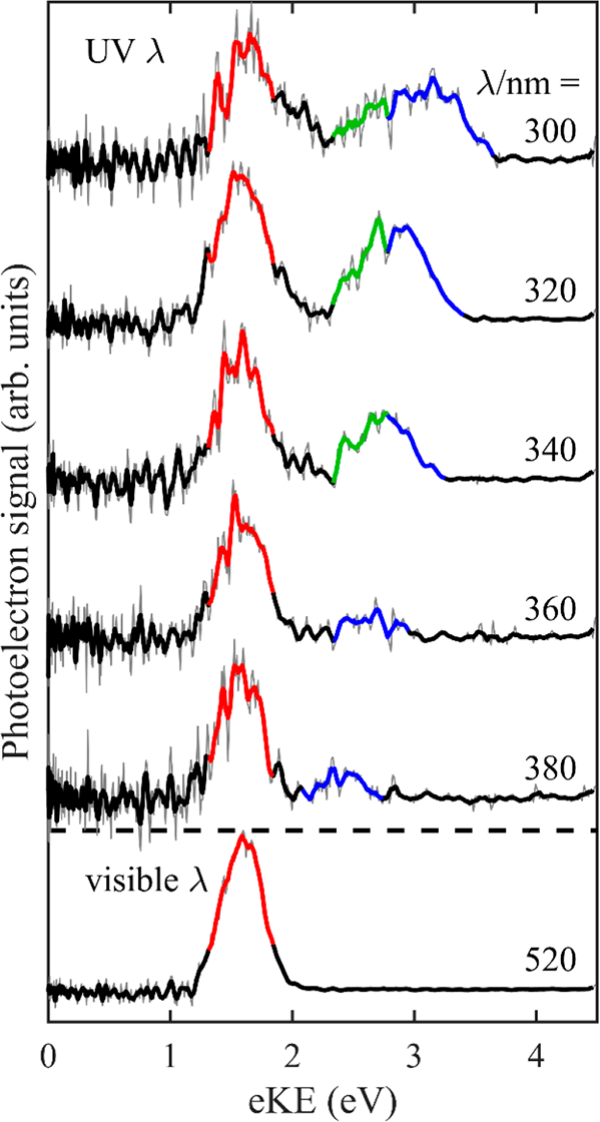
Photoelectron spectra of Fl^2–^ recorded in the range λ = 300–380 nm and at λ = 520 nm. The gray lines are the raw data, and the black lines are a five-point moving average. The red line highlights tunnelling via RCB_1_, the green line highlights tunnelling via RCB_2_, and the blue line highlights direct detachment over the lowest RCB.

**Figure 3:**
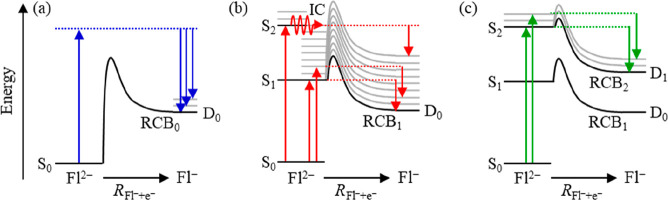
Schematic energy level diagram of Fl^2–^ and Fl^–^ and relevant photoemission processes. (a) Direct detachment over the lowest energy barrier, RCB_0_ (highlighted in blue in Figure 2); (b) tunnelling through RCB_1_ from S_1_ following excitation to either S_1_ directly or to S_2_ that subsequently internally converts (IC) to S_1_ (highlighted in red in Figure 2); and (c) tunnelling through RCB_2_ from S_2_ (highlighted in green in Figure 2). Upward arrows indicate excitation and downward arrows electron emission. Dashed lines highlight the energy imparted following excitation. The gray lines indicate vibrational levels with associated RCBs for relevant electronic excited states.

The photoelectron spectrum at λ = 520 nm shows the resonant tunnelling through the S_1_ state RCB that has been considered previously.^11^ With reference to Figure 3b, the eKE of 1.64 eV corresponds approximately the energy gap between S_1_ of the dianion and D_0_ of the final anion state. As the S_1_ state lies 2.45 eV (507 nm transition energy, Figure 1) above S_0_, the energy of D_0_ lies approximately 2.45 – 1.64 = 0.81 eV above S_0_, which is consistent with the VDE (∼0.7 eV) determined from the direct detachment peak. The feature at eKE = 1.64 eV persists also in the UV spectral range (highlighted in red in Figure 2), where the excitation is resonant with the S_2_ ← S_0_ transition (see Figure 1). Therefore, at least some population is following Kasha’s rule. As shown schematically in Figure 3b, excitation to S_2_ can lead to IC to populate S_1_ with a large amount of internal energy, and as an electron subsequently tunnels through the S_1_ state RCB (RCB_1_), internal energy will be conserved in D_0_, producing the same eKE spectrum peaking at eKE = 1.64 eV. This Kasha behavior was also observed in the bisdisulizole tetra-anion.^36^

We now turn to the third feature in Figure 2, the low-energy edge to the direct detachment peak, which does not shift with eKE (highlighted in green). This edge does not correspond to a direct detachment cutoff because there is no RCB at this energy for detachment to the D_0_ state. If there was, then the direct detachment peak (highlighted in blue) would not be visible in the λ = 380 nm spectrum (or the 400 ≤ λ ≤ 440 nm spectra shown previously).^11^ Instead, we consider the possibility that this feature arises from resonant electron tunnelling through the RCB of the S_2_ excited state (RCB_2_). The feature peaks approximately at eKE ≈ 2.5 eV (although it is difficult to ascertain exactly because of the spectral overlap with the direct detachment highlighted in blue). The S_2_ state lies at 3.82 eV (325 nm from Figure 1), and using a similar energetic argument as for S_1_ resonant tunnelling, we find that the final D_*n*_ state in the anion lies approximately 3.82 – 2.5 = 1.32 eV above the S_0_ state. This is clearly inconsistent with the D_0_ state of Fl^–^, which we found to be around ∼0.8 eV above S_0_. However, an excited state in Fl^–^ following resonant electron tunnelling through RCB_2_ could potentially be the final state.

The electronic structures of Fl^2–^ and Fl^–^ were considered using computational chemistry, with the results summarized in Table 1. All energies for Fl^2–^ are quoted relative to S_0_ and are considered as vertical excitations (*i.e*., in the S_0_ geometry). For Fl^–^, both vertical and adiabatic energies are quoted, and the D_1_ excited state is considered in the D_0_ geometry. The calculated transition energies for the S_1_ and S_2_ states are in reasonable agreement with the fragment action spectrum (Figure 1). Similarly, the calculated VDE is in good accord with that obtained from the photoelectron spectrum (Figure 2). Hence, the calculations have captured the essential electronic structure. On the basis of our proposed resonant tunnelling through RCB_2_, we anticipate an excited state of the anion around 1.32 eV, and our calculations indeed show that the D_1_ state lies at 1.35 eV. The detachment dynamics can be schematically represented as shown in Figure 3c. But why would resonant electron tunnelling from S_2_ lead to D_1_ rather than D_0_?

**Table 1:** Calculated Energetics with VDEs and VEEs Calculated using DFT and TD-DFT (TDA) B3LYP/6-311G ++ 2d,2p and Experimental Values Extracted from the Fragment Action Spectrum (Figure 1) and Photoelectron Spectra (Figure 2)^a^

species	calculated vertical energy	experimental energy
Fl^2–^ (S_0_)	0	0
Fl^–^ (D_0_)	0.74 (ADE 0.62)	0.7
Fl^–^ (D_1_)	1.35	1.32
Fl^2–^ (S_1_)	2.66	2.3
Fl^2–^ (S_2_)	3.01	3.6

a All energies are relative to S_0_ and in electronvolts.

It is convenient to think of the first excited state as arising from the promotion of an electron from the highest-occupied molecular orbital (HOMO) to the lowest-unoccupied molecular orbital (LUMO) and subsequent excited states to promotion to successively higher-lying LUMOs (e.g., LUMO+1, LUMO+2, etc.). In a Koopmans’ picture,^37^ this would form the same anion state from Fl^2–^ whether tunnelling occurred via RCB_1_ or RCB_2_. However, higher-lying states often have mixed character, including core-excited character. To assess this possibility, we considered the electron configurations of the relevant electronic states in terms of the relevant molecular orbitals of Fl^2–^: HOMO, HOMO–1, LUMO, and LUMO+1 (labeled henceforth as **1**, **2**, **3**, and **4** with increasing energy). The results of the calculations indicate that, in a Koopmans’ picture, the removal of the electron in the highest-lying MO from the S_0_ (**1**^2^**2**^2^) or S_1_ (55% **1**^2^**2**^1^**4**^1^ and 45% **1**^2^**2**^1^**3**^1^) states results in the electronic configuration of the D_0_ (**1**^2^**2**^1^) state. The S_2_ (60% **1**^1^**2**^2^**3**^1^ and 31% **1**^1^**2**^2^**4**^1^) and D_1_ states (97% **1**^1^**2**^2^) have predominantly core-excited character, such that electron loss from the S_2_ state would result in the D_1_ state in a Koopmans’ picture. Hence, the calculations fully support our interpretation that the feature in Figure 2, highlighted in green, arises from resonant electron tunnelling through RCB_2_, which is associated with the S_2_ electronic state.

Finally, additional evidence that two distinct detachment channels contribute to the high eKE feature for λ < 360 nm can be gained from the electron angular distributions. These are generally quantified by an anisotropy parameter, −1 ≤ β_2_ ≤ +2, where β_2_ < 0, = 0, and > 0 correspond to emission predominantly perpendicular, isotropic, and parallel to the laser polarization, respectively.^38^ Abrupt changes in β_2_ are indicative of changes in electronic character of the molecular orbital from which the electron was removed.^28,39,40^ Across the high-eKE feature, the green and blue highlighted areas have distinct β_2_ parameters. Specifically, the part assigned to tunnelling though RCB_2_ (green) has β_2_ ∼ 0, while that for direct detachment (blue) has β_2_ ∼ −0.5, confirming the two distinct electron loss channels. The tunnelling feature through RCB_1_ (red) has β_2_ ∼ −0.2, in agreement with the previously reported value.^11^

On the basis of the above, we conclude that resonant electron tunnelling occurs from both the S_1_ and the S_2_ states. However, why are both pathways accessible at the same photon energy? For both to be present simultaneously, IC from the S_2_ to the S_1_ state followed by tunnelling through RCB_1_ (Figure 3b) must be competitive with electron loss via tunnelling through RCB_2_ (Figure 3c). While we were not able to directly measure the tunnelling rate through RCB_2_ in real time, the current experiments do provide some clues into the relative time scales of different processes. As anisotropic photoelectron angular distributions indicate tunnelling occurring on a time scale shorter than rotational dephasing,^41^ the isotropic RCB_2_ tunnelling feature (green) compared to the anisotropic RCB_1_ tunnelling feature (red) suggests that resonant tunnelling through RCB_1_ may be faster than through RCB_2_. In addition, our previous time-resolved measurements on Fl^2–^ found the tunnelling lifetime through RCB_1_ to be ∼1 ps,^11^ which is significantly faster than has been observed in many other systems.^13−17^ Therefore, even if tunnelling via RCB_2_ is slower than via RCB_1_, it may still be relatively fast. It has also been argued that IC from the S_2_ state in Fl derivatives may be relatively slow because of the large S_1_–S_2_ gap (∼0.7 eV) and the large differences in the localization of the electron density in both excited states.^26,42^ Therefore, given the observation of non-Kasha’s rule electron loss, it seems likely that tunnelling through RCB_2_ occurs on a similar time scale to IC from S_2_ to S_1_ and that both processes are slower than tunnelling via RCB_1_ (>1 ps).

For the other polyanionic systems where resonant tunnelling has been observed,^13−17^ IC will likely outcompete electron emission, resulting in photoelectron emission that appears to adhere to Kasha’s rule. This seems to be the case in the bisdisulizole tetra-anion^12^ and in the protoporphyrin IX dianion.^20^ We also note that Kappes and co-workers have observed competitive electron tunnelling from the lowest singlet, S_1_, and triplet, T_1_, states in the isolated Pd^(II)^*meso*-tetra(4-sulfonatophenyl)porphyrin tetra-anions.^15^ The observation of both S_1_ and T_1_ electron emission then requires that the rate of intersystem crossing relative to that of tunnelling is competitive. Finally, it should be noted that anti-Kasha’s fluorescence in the condensed phase has been observed previously.^42,43^

The overall picture of a single electronic RCB in a polyanion is clearly invalid, and the energetic landscape is not only spatially anisotropic, but it is also highly complex in energy with an RCB for each (ro)vibrational level of each electronic state. Additionally, not every electronic state in the dianion will correlate to the same electronic state in the final anion following removal of an electron, although a Koopmans’ picture seems to hold for electron tunnelling. Note that we have solely considered the electronic RCBs and furthermore note that there are also RCBs associated with fragmentation, which will further complicate the picture.^44,45^ The multitude of electronic RCBs can stretch many electronvolts above the lowest direct detachment channel. In the present case of Fl^2–^, we observe non-Kasha rule behavior either because the IC rate from S_2_ to S_1_ is relatively slow or because the tunnelling through the RCB is relatively fast. Generally, it is the competition between these processes that determines whether Kasha’s rule will be followed. On the basis of the observation that in most cases Kasha’s rule is followed, resonant electron tunnelling through high-lying excited state RCBs is not necessarily as fast (or at least significantly slower than IC) as one might intuitively expect.

## Notes

Data is available from the corresponding authors upon reasonable request.
